# RETRACTED ARTICLE: Learnings from Industry 4.0 for transitioning towards Industry 4.0+: challenges and solutions for Indian pharmaceutical sector

**DOI:** 10.1007/s10479-023-05391-6

**Published:** 2023-05-26

**Authors:** Mahak Sharma, Rajat Sehrawat, Mihalis Giannakis, Yogesh K. Dwivedi

**Affiliations:** 1 Department of Industrial Engineering and Business Information Systems (IEBIS), Faculty of Behavioural, Management and Social Sciences (BMS), University of Twente, Enschede, The Netherlands; 2 Booking.com, Amsterdam, The Netherlands; 3 Audencia Nantes Business School, 8 Route de La Jonelière, B.P. 31222, 44312 Nantes Cedex 3, France; 4 Digital Futures for Sustainable Business and Society Research Group, School of Management, Swansea University, Bay Campus, Fabian Bay, Swansea, SA1 8EN Wales, UK; 5 Department of Management, Symbiosis Institute of Business Management, Symbiosis International (Deemed University), Pune, Maharashtra India

The Editor-in-Chief and the publisher have retracted this article. The article was submitted to be part of a guest-edited issue. An investigation by the publisher found a number of articles, including this one, with a number of concerns, including but not limited to compromised editorial handling and peer review process, inappropriate or irrelevant references or not being in scope of the journal or guest-edited issue. Based on the investigation's findings the Editor-in-Chief therefore no longer has confidence in the results and conclusions of this article.

The authors disagree with this retraction.

The online version of this article contains the full text of the retracted article as Supplementary Information.

## Supplementary Information

Below is the link to the electronic supplementary material.

Supplementary file1 (PDF 960 KB)

