# Supplementary material for: RETRACTED ARTICLE: Learnings from Industry 4.0 for transitioning towards Industry 4.0+: challenges and solutions for Indian pharmaceutical sector
Source: Ann Oper Res. 2023 May 26;337(Suppl 1):27–8. doi: 10.1007/s10479-023-05391-6 (PMC10214346; doi:10.1007/s10479-023-05391-6)
Supplement: Supplementary file 1 — Supplementary file1 (PDF 960 KB) [file 10479_2023_5391_MOESM1_ESM.pdf]

Annals of Operations Research
https://doi.org/10.1007/s10479-023-05391-6

ORIGINAL RESEARCH

Learnings from Industry 4.0 for transitioning towards Industry 4.0+: challenges and solutions for Indian pharmaceutical sector

Mahak Sharma[1] · Rajat Sehrawat[2] · Mihalis Giannakis[3] · Yogesh K. Dwivedi[4,5]

Accepted: 9 May 2023
© The Author(s) 2023

RETRACTED ARTICLE

Abstract

Industry 4.0 (I4.0) is helping production units to become smarter using cyber-physical systems and cognitive intelligence. The advanced diagnostics with I4.0 technologies (I4.0t) help in making the process highly flexible, resilient and autonomous. Still, the adoption of I4.0t especially in emerging economies like India is at a very slow pace. The present research has used an integrated approach i.e., Analytical Hierarchy Process-Combinative Distance-Based Assessment-Decision-Making Trial and Evaluation Laboratory to propose a barrier solution framework using data from pharmaceutical manufacturing sector. The findings reveal that "Costly venture" is found to be the most critical deterrent while "Customer awareness and satisfaction" is one of the potential solutions for I4.0 adoption. Further, lack of standardisation and fair benchmarking policies especially in the context of developing economies needs immediate attention. This article concludes by proposing a framework which will help to move from I4.0 towards Industry 4.0 + (I4.0+) which emphasises on role of collaboration between man and machine. And leads to sustainable supply chain management.

✉ Yogesh K. Dwivedi
y.k.dwivedi@swansea.ac.uk

Mahak Sharma
mahaksharma[illegible]@gmail.com

Rajat Sehrawat
rajatsehrawat91@gmail.com

Mihalis Giannakis
[illegible]giannakis@audencia.com

1 Department of Industrial Engineering and Business Information Systems (IEBIS), Faculty of Behavioural, Management and Social Sciences (BMS), University of Twente, Enschede, The Netherlands

2 Booking.com, Amsterdam, The Netherlands

3 Audencia Nantes Business School, 8 Route de La Jonelière, B.P. 31222, 44312 Nantes Cedex 3, France

4 Digital Futures for Sustainable Business and Society Research Group, School of Management, Swansea University, Bay Campus, Fabian Bay, Swansea SA1 8EN, Wales, UK

5 Department of Management, Symbiosis Institute of Business Management, Symbiosis International (Deemed University), Pune, Maharashtra, India

Keywords Industry 4.0 · Supply chain performance · Sustainable supply chain management · Developing economies

1 Introduction

The fierce competition and demand for Sustainable Supply Chains (SSCs) have pushed firms to streamline their manufacturing structures to a smart level. A manufacturing process is considered smart if it comprises of intelligent, agile, well equipped, and flexible manufacturing systems to encounter the obstacles of a dynamic market (Moeuf et al., 2020). Manufacturers all over the world are facing resource scarcity due to eccentric resource utilization practices and unsustainable manufacturing (Zheng et al., 2021). A lack of visibility in performing accurate sales forecasting may lead to losing customer orders which surely cause reduction in customer base thus reducing profit margins (Bag et al., 2021). Hence, for the development of knowledge economy and its intellectual component, innovation development and advancements in socio-economic indicators can be proven as the growth vector of Industry 4.0 (I4.0) in near future (Bogoviz et al., 2019; Nahavandi, 2019).

I4.0 introduces new opportunities that may disrupt the traditional approach of manufacturing firms by providing enhance visibility and better customisation (Bag et al., 2021; Sharma et al., 2022a, 2022b). Industries like pharmaceutical where affordable personal customisation can save millions of life and virtual simulation can save lot of investment, I4.0 technologies can act as a disrupting force. Since I4.0 can break down the barriers between the real-world and virtual worlds (Aslam et al., 2020), augmented reality (AR) can be used in people's everyday life, especially in health care sector. Also, prior literature has clearly emphasized that the potential advantages of I4.0 have not been used in the pharmaceutical industry since it is expected that combination of AI and cloud computing will initiate faster process flows with high accuracy and comparatively lower costs.

I4.0 is a manufacturing revolution which is significantly impacting governance, society, and economy and will also help to achieve sustainable development goals (Fareri et al., 2020). I4.0 emphasizes on the need of digitalization and AI-driven technologies for enhancing the productivity and elasticity of production (Xu et al., 2021). Firms has to overcome multiple impediments while moving towards digitalisation; however, if this migration will lead to maximisation in monetary benefits is still not evident from the existing literature. Hence, it is critical to understand the influence of smart manufacturing on environmental economic and sustainability aspects (Nara et al., 2021) which demands an in-depth investigation (Sharma et al., 2021). It is also for utmost importance to consider the social impacts of I4 such as the replacement of human beings by machines and new technologies (Sharma et al., 2022a, [illegible]b). However, on the contrary, these emerging technologies will surely lead to better working conditions such as decentralised decision-making and improved safety (Zheng et al., [illegible]). I4.0 provides enhanced visibility to overcome the challenges that impede implementation of proper digitalised delivery system (Bag et al., 2021). However, in case of emerging technologies, there lies a large chasm in identification of barriers and proposing the most appropriate solution from the set of potential solutions (Makkonen, 2021).

India is excellent at manufacturing things ranging from pin to plane and from daily use-products to high-tech gadgets.[1] I4.0 technologies (I4.0t) can help India to reinvent its manufacturing space and move up in the value chain. Indian economy is expecting significant

[1] https://hbr.org/2015/09/understanding-the-rise-of-manufacturing-in-india.

benefits from I4.0t in augmenting the manufacturing sector's share by 25% of its GDP[2] by 2022. However, being a developing economy, investing in emerging innovations can be risky which can adversely impact the overall growth. Most of the firms believe that barriers such as high investment requirements, lack of adequate skills in workforce, uncertainty about economic benefits, cyber-security challenges, lack of infrastructure and poor value-chain integration can lead to disaster.

The standard feature of an I4.0 emphasizes on the integration of connectivity, artificial intelligence (AI), and robotics to enable real-time and online data availability and optimize manufacturing and enterprise-wide management (Arden et al., 2021). In pharmaceutical manufacturing, external information—including variables such as patient experience, market demand, supplier inventories, and public health emergencies—could fuse with internal information such as energy and resource management, modeling and simulation outcomes, and laboratory data. Integrating internal and external data sources enables unprecedented real-time responsiveness, monitoring, control, and prediction which will not only improve efficiency of system but also provide customised products and customer delight experience. I4.0 will result in a well-controlled, hyper-connected, digitized ecosystem and pharmaceutical value chain.

There is a need for research that can propose potential and suitable solutions for such impediments. It is seen over the last few decades that removing misconceptions using prior research and taking calculated risks is the way most of manufacturing firms work in India.[3] However, no study till date has used a detailed method to identify barriers and examine suitable (potential) solutions that will lead to SSCM. Lack of empirical evidences demands for novel research before reaching any universal decision (Sharma et al., 2022a, 2022b). I4.0 adoption framework has been proposed in previous research works (Frank et al., 2019). However, there still lies a foramen which needs thorough investigation i.e., if organisations need to examine impact of I4.0 and human–machine interaction. Since I4.0 emphasises on digitalisation, this is a step forward (I4.0 +) where firms are advised to use human expertise to fetch better results from machines. It is also expected that collaboration between man and machine may lead to better synergies and promising outputs. Hence, the authors propose the following research questions:

RQ1: What are the barriers to I4.0 adoption in the pharmaceutical sector of India?

RQ2: Which barriers are critical to I4.0 in the pharmaceutical sector of India?

RQ3: What can be the effective Potential Solutions (PSs) for I4.0 adoption in the pharmaceutical sector of India?

RQ4: What are the cause and effect relation among the barriers?

RQ5: What are the cause and effect relation among potential solution practices?

The rest of this paper is organised as follows: Sect. 2 first explains methodology followed for conducting the literature review. Further this section discusses Industry 4.0 (I4.0) from the focal lens of digital transformation and sustainable solutions. Section 2 concludes by summarising the identified research gaps. Section 3 discusses the research methodology while Sect. 4 demonstrates the analysis and findings of the work. Discussions are presented

2 https://economictimes.indiatimes.com/news/company/corporate-trends/covid-just-gave-industry-4-0-the-push-it-needed-in-india/articleshow/85392907.cms?from=mdr.

3 https://webapps.itc.utwente.nl/librarywww/papers_2016/phd/sengupta.pdf.

RETRACTED ARTICLE

in Sect. 5 where the authors have also proposed an advanced I4.0 framework. Further this section also provides both academic and practical implications. The article concludes in Sect. 6 where we have also presented the limitations as well as proposed directions for future research.

2 Literature review

Recent studies provide a healthy foundation for exploring I4.0 and its impact on sustainable supply chain management. I4.0 is currently very critical for many universities, research centers, practitioners in the organisations, yet the academicians and subject experts believe that the I4.0 term itself is unclear (Ghobakhloo, 2018), hence making it rather difficult for manufacturing firms to understand the phenomenon. The present scenario also makes it difficult to identify the stages needed for the transition toward I4.0. Also, with the evolving customisation needs and firms trying to move towards semantic interoperability while focussing on human role, it is important to identify the stages needed for the transition toward I4.0+. Hence the author has done a systematic literature review to analyse the phenomenon from different angels such as with respect to technology trends, human–machine interaction, and early fault detection with Internet of Things (IoT). The authors have explored how the innovative digital technologies collectively facilitate the growth of I4.0 i.e., the latest digital industrial revolution (Liao et al., 2017) and also investigated the challenges faced by firms while adopting I4.0 (Kamble et al., 2018a, 2018b).

2.1 Industry 4.0: digital transformation and sustainable solutions

I4.0 is the "*new industrial stage*" where manufacturing operations systems are modified with the help of information and communication technologies (ICT) (Dalenogare et al., 2018). I4.0t helps in integration of architecture manufacturing with information technology (Bai et al., 2020). I4.0 also considers the exchange of real-time information for better integration of the supply chain (Bogoviz et al., 2019). I4.0t are classified into physical technologies (drones, smart sensors, additive manufacturing) and digital technologies (big data, blockchain, machine learning (ML), robotics, cloud computing, radio-frequency identification (RFID), artificial intelligence (AI), augmented reality and the Internet of Things (IoT).

Pharmaceutical companies constantly struggling towards finding innovative methods that can provide affordable personalised treatments that help people live longer and healthier lives. The manufacturing control system of pharmaceutical production needs to be flexible and self-organized in accordance with external market's demands where I4.0t can contribute and streamline the processes. I4.0 is expected to improve operational performance in the entire healthcare sector (Wan et al., 2018). It is also expected that digital transformation with I4.0t will have significant consequences on sustainability (de Sousa Jabbour et al., 2018; Kamble et al., 2018a, 2018b). However, there have been no study till date that has explored the implications of I4.0 and manufacturing digitisation for sustainable development in context of Indian pharmaceutical sector which demands further exploration.

I4.0 is a revolutionary innovation that is disrupting and transforming the processes across all business and manufacturing verticals by bring together the holistic power of technology with new industrial prototypes. As the current production systems are antediluvian and cannot accomplish as per the ever-changing buyer requirements. Also, the traditional model (take-make-dispose) of linear economy is unable to utilise the non-renewable natural resources

RETRACTED ARTICLE

judiciously. This societal and environmental imbalance is disturbing the ecological sustainability and having negative effect on the economic conditions. Since, the entire manufacturing process is not sustainable and environment friendly there is a need of substantial measures to increase productivity, the quality of manufacturing, decrease error rates, and reduce waste production. The present viable solution is smart manufacturing that stimulates an augmented automation (Kamble, et al., 2018a, 2018b; Luthra & Mangla, 2018) where robots can perform tasks with more precision hence significantly reducing the error rate and increasing productivity (quality and quantity). I4.0 represents a smart manufacturing networking process where interaction between machines and products is carried out without human control (Ivanov et al., 2019). Using CPS principles, I4 empowers flexible production process strategies as well as designs that help in producing highly customised goods (Kamble et al., 2018a, 2018b; Kumar et al., 2016). The precise real time operation sequencing can be easily achieved with smart sensors and plug and produce CPS for better capacity utilization (Ivanov et al., 2019; Theorin et al., 2017). Digital technologies related to I4.0 like big data analytics and blockchain facilitate active forecasting and effective management of risk to enhance the resource reconfiguration capability (Ivanov et al., 2019). Firms in manufacturing domain are transforming in terms of time and space flexibility, with more transparent, energy-efficient and decentralized systems (Horváth & Szabó, 2019). Using the real time data exchange, AI expedites effective interconnection among processes, machines and goods that enables rapid decision-making. The latest production units facilitate manufacturing activities with learning and interactive autonomous robots (Horváth & Szabó, 2019; Müller et al., 2018). The digitalized process focusses on value creation, value capture and value offer (Müller et al., 2018) thereby helping production units to build up their relationships with different stakeholders and customers. This digital transformation in manufacturing domain demand significant amendments. Though servitization of production systems will simplify few processes but the chances of making other processes more complex cannot be ignored. Segal (2018) also forecasted that automation risks more than 45% jobs all over the world. It is expected that the digitised production systems demand novel skills from workforces which may lead to substantial decline in positions requiring lower credentials thereby causing employment disruptions. Another school of thought emphases that I4.0's effective adoption will be an amalgamation of social appropriateness as well as industrial viability. I4.0 may also slow the pace of the relocation of manufacturing processes to emerging economies owing to problems related to standardization of mass products (Ancarani & Di Mauro, 2018). Firms are using I4.0 and related technologies as a solution to the ever-enhancing race in manufacturing domain forcing production units to have quicker time-to-market, enhance productivity and have shorter product lifecycle (Sharma & Sehrawat, 2020a, 2020b; Sharma et al, 2020a, 2020b, 2020c). Hence, to enjoy long-term competitiveness, firms especially in emerging economies are looking towards I4.0 as a way that will help them to endure and even excel in the evolving environment. Rajput and Singh (2019) has proposed that an integrated Circular economy (CE) and I4.0 based supply chain will be highly embedded, more sustainable, flexible, secured, self-organized, and interoperable. It is also proposed that I4.0 can serve as a key innovative step towards the transformation of the manufacturing sector from linear to CE where blockchain can serve as the driving force (Kshetri, 2018). Furthermore, there is a dearth of studies that compare impact of I4.0 on different economies. Li (2017) compared barriers with respect to China's "Made-in-China 2025" and Germany's "Industry 4.0". Raj et al. (2020) analysed adoption barriers of I4.0 and their cause-effect relationships for manufacturing firms located in India and France.

The major challenges barricading the adoption of I4.0 are technological integration and synchronization; high initial implementation cost, long gestation period before return on

RETRACTED ARTICLE

investments, data security and ownership concerns, low degree of standardization, lack of skilled personnel, additional investments and time on training of existing workforce (Hofmann et al., 2017; Kiel et al., 2017; Müller et al., 2018; Sung, 2018). There have been other deterrents to I4.0 such as lack of consumer awareness, standardization, concerns about cybersecurity, and poor understanding of integration are also deterrent to I4.0 (Ahsan and Rahan 2017; Ivanov et al., 2019; Machado et al., 2020). It is very critical that government or related stakeholders work on the enhancement of public recognition and end-customers education with regard to the importance of reducing waste, using the proper methods of disposal and opting for sustainable solutions. Further, it is also imperative to emphasize the responsibility of privacy-ensuring techniques that defend user information and privacy are also not standardized across globe. Further, concerns related to the development of manufacturing structures also substantially influence the fragility risk, thereby creating uncertainties in the environment (Horváth & Szabó, 2019). Firms especially in emerging economies have not yet investigated factors, developed business cases and conducted feasibility studies that can capture the pain points related to I4.0 (Basl, 2017; Horváth & Szabó, 2019). Further, factors like cultural acceptance and organizational resistance are also sparsely studied in the existing literature related to I4.0 (Kamble et al., 2020; Machado et al., 2020).

The technological competitiveness and the emerging technologies of I4.0 have helped to increase organizational competition and excellence in all sectors and industries. I4.0 focussed on improvement of the production environment by adopting digitalisations and this technological competitiveness leads to promote organizational performance and sustainability. Many studies have proposed that organizational competitiveness as one of the primary pillar for adoption of I4.0. On the other hand, there is a lack of research that presents the advantage of implementing technological competitiveness and the emergence of I4.0 as a key to revolutionizing the landscape of goods production in various sectors. Several recent studies explored case studies from different countries highlighting I4.0 benefits. Bhatia and Kumar (2020) identified critical success factors (CSF) for Industry 4.0 and also studied the relationships between CSF and performance outcomes using regression analysis. The findings indicate that "Data governance" and "Legal aspects" are critical in achieving performance outcomes about I4 technologies in the automotive manufacturing industry. Aslam et al. (2020) proposed an innovation framework, "Absolute Innovation Management (AIM)" to help the prospective adopters in understanding, implementing, planning corporate strategy in synergizing the innovation ecosystem. This framework also guides for design thinking which may help businesses to get ready for IoT and the I4.0 revolution to achieve competitive advantage and economic growth. However, Rauch (2020) and Skobelev & Borovik (2017) stated that I4.0 is not limited to the manufacturing sector. They believe I4.0 can solve social problems by using these advanced IT technologies, IoT, robots, and artificial intelligence (Skobelev & Borovik, 2017). In 2016, the Japanese Council for Science, Technology, and Innovation; and the Japanese Cabinet Office promoted the use of the term I4.0 as this term was coined by Japan's most important business federation, Keidanren. There are also a handful of studies that have proposed a cyber-physical-based PAT framework called CPbPAT for implementing smart manufacturing systems in the pharmaceutical industry to examine how CPbPAT responds to unexpected changes.

RETRACTED ARTICLE

3 Research gaps

In this present era firms should look for ways to accomplish societal goals, where human and machine work in symbiosis. The firms look for ways that can arm them better against disruptions, ensures higher degree of robustness, and offer infrastructure in times of crisis. Interconnection and combining the strengths of human and machine lead to an actionable intelligence (Xu et al., 2021). I4.0+demands to look beyond I4.0 practices where firms look for sustainable solutions by placing planet before firm growth vector, place employee wellbeing before mass-production, provide safe and inclusive work environment, find agile and resilient alternatives with adaptable and flexible technologies (Xu et al., 2021). Hence, the need of the hour to meticulously look for ways that provides technology-driven benefits but keeping society-centric and human-centric approaches in the heart of production units. Hence, firms conceptualize to leverage the inimitable imagination of human to join forces with precise, smart and powerful technology (Maddikunta et al., 2022). Till date no research has proposed a human–machine collaboration framework that can assist practitioners to understand the functioning of I4.0. This study tries to explore the relevant choices and proposes a framework that can help a firm to move towards I4.

Further, researcher have widely used various techniques especially Multi-Criteria-Decision-Making (MCDM) for emerging technologies like I4.0 (Sharma et al., 2021). Only a handful of research works have tried to explore Sustainable aspect of SCM (SSCM) and I4.0 (de Sousa Jabbour et al., 2018; Rajput et al., 2019; Yadav et al., 2020), operations (Mittal et al., 2018), logistics and lean manufacturing, and big data analytics and additive manufacturing (Ivanov et al., 2019) perspectives but there are no such studies that have provided barrier-solution framework for pharmaceutical firms for implementing I4.0.

4 Research methodology

For purpose of conducting this research, we have implemented Modified grounded theory (SGT) approach (Strauss & Corbin, 1990) to investigate the barriers and propose potential solutions influencing the implementation of I4.0. SGT uses three steps iterative coding of qualitative data. In this research, qualitative data was collected in form of interviews from industry experts from pharmaceutical firms and academic experts who had knowledge of manufacturing in pharmaceutical domain. The iterative data collection methodology spans over four stages. In the first stage, an exhaustive literature review followed by professional interviews have been conducted. In the second stage, the professionals were asked to prioritise the barriers using AHP (Saaty, 2008) to rank them as per their criticality. In the third stage, the cases were closely studied, and SIs have been proposed as per the suitability and requirement of each case which were ranked by experts using COmbinative Distance Based ASsessment (CODAS) (Keshavarz Ghorabaee et al., 2016). In the present study, the priority weights from AHP have been used as initial inputs for CODAS approach to calculate the decisive ranks of the SIs. In the fifth stage, experts were requested to fill the comparative matrix using DEMATEL scale (Gabus & Fontela, 1973) for computing the cause-effect relationships among barriers and SIs respectively.

The selected Indian firms are leading pharmaceutical manufacturing firms in India. Firm A established in 1989. The case organisation has a workforce of over 600 employees (641 in 2022) and a net income of US$ 15 million (2022). Firm B was established in 2018 with a

RETRACTED ARTICLE

workforce of over 200 (213 in 2022) employees. The production units chosen for the investigation encountered problems related to I4.0 solutions implementation. Also, the firms want to focus on how human–machine collaboration can pave a path to move towards excellence. Firm A has already adopted few I4.0 technologies (cloud computing, big data) and are progressing for IoT, additive manufacturing robotics system and augmented reality. Firm B is also very progressive in terms of technology adoption and is transitioning towards implementation of IoT. 20 Experts from Firm A and B have been interviewed to understand the problem in detail. The clear understanding of impediments plays a vital role in outlining schemes by managers' and top officials' decision making for smoother I4.0 adoption. Further 14 experts from Firm B have helped in filling the comparison matrices (Refer Table 5 in Appendix).

5 Analysis and results

This section provides analysis for all stages in a sequential manner where we first present qualitative and then quantitative results. The findings from the interviews have been discussed in qualitative analysis subsection while results after applying integrated methodology have been elaborated in quantitative analysis subsection.

5.1 Stage 1—qualitative (interview) analysis

To answer the research question i.e., "***What are the barriers to I4.0 adoption in the pharmaceutical sector of India***", *and* "***What can be the effective potential solution (PSs) for I4.0 adoption in the pharmaceutical sector of India?",*** the authors have done an extensive literature review followed by iterative coding of the interviews that helped in identification of comprehensive examination of relevant barriers (Refer Table 1) as well as probable potential solutions (Refer Table 2).

The inductive coding scheme has been used to explore the factors/constructs (barriers as well as potential solutions). All the authors have separately read the interview transcript (see Appendix) and proposed coding scheme. The next step was to merge similar code and propose actual constructs. Most of the interviewees have emphasized on the need for heavy investment in technologies that has emerged as an important deterrent named as "costly venture". Further the management in the organisation was also sceptic about the issues related to data masquerading and lack of support from different stakeholders including supplier as well as employees.

The present research tries to propose a comprehensive list of potential solutions that will help an organisation to look towards I4.0 from the focussed focal lens where one can weigh if benefits outweigh barriers. Most of the interviewees have emphasized need for "Customer awareness and satisfaction" in order to adopt such solutions at organisational level. This can only be achieved if seminars or workshops can be arranged to educate customers and explain the benefits of technology, recycling practices and sustainability. Further, few interviews also proposed to have advertisements and right branding in order to have national as well as international intention to move towards I4.0. The next critical factor that can promote adoption of I4.0t is "Organisational/institutional awareness". This will help the organisation to formulate right and timely policies.

RETRACTED ARTICLE

Table 1 Barriers impeding the adoption of I4.0

Barriers	Definition	Source
Costly venture	It includes investment on prototype robots that can mimic human and can sense and analyse human intention, pick to light system and human–machine interface. Increase in almost 50% per capita yearly investment to re-engineer existing strategies. It includes investment on Intelligent and vibration sensors, and pick to light system	Geissbauer et al. (2014), Rajput and Singh (2019), Sharma et al. (2022a, 2022b)
Infrastructure standardisation	Infrastructure needed to prepare autonomous functioning and integration of heterogeneous components using advanced diagnostics such as big data and IoT	Leitão et al. (201[illegible]), Xu et al. (2018)
Automation and digitisation of process	Intertwining among digital and physical process helps in better inventory management and operations. This convergence helps in establishing global networks that integrate warehouse systems, machinery and manufacturing/production facilities	Xu et al. (2018), Horváth & Szabó (2019)
Value chain integration's challenges	Human has the tendency to resist change hence an organisation need to look for means that can assist its employees and also other employees in various firms in the value chain to understand the importance of migration	Sharma et al., (2022a, 2022b)
Ineffectiveness in change management	Ineffective management of changing processes executives, employees, value chain members and third party channel partners. The introduction of advanced technologies, which are way ahead of conventional methods of manufacturing are resisted by personnel with limited or traditional skill set	de Sousa Jabbour et al. (2018), Sharma et al. (2020a)

RETRACTED ARTICLE

Table 1 (continued)

Barriers	Definition	Source
Counterfeit and absence of correct information	Wrong and untimely information may lead to disaster and may sometimes hit the company image. The company should know when and where to share the correct information	Stegemann (2016)
Lack of simple and fair benchmarking	In order to standardize the performing systems, a company needs rational and unbiased assessment systems which can correctly predict the future of sustainable solutions	Ding (2018)
Standardisation, Regulations, Legalisation and Certification issues	There is a lack of system-level perspective, international cooperation efforts, set of uniform technical standards, reference architecture model, Industrial Internet Reference Architecture	Stegemann (20[illegible]), Ding (2018), Xu et al. (2018)
Employment disruption (Skill gaps and need for additional trainings etc.)	Requirement of new skills and trainings for job continuation/retention	Sharma et al., (2022a, 2022b)
Automation Errors	Inappropriate data modelling, feedback or over automation; error in correctly realising/assigning required task; or due to dissonance control for human reliability in CPS	
Disproportion between technology development, social evolution	The firms are moving to new technology paradigm and progressing towards smart manufacturing but completely/partially ignoring its impact on society and environment	Author's own definition
Poor openness, accessibility, and availability	System crash, uncoordinated interaction, errors due to problems in updated data at server, inaccurate data transfer, power failure, may lead to problems related to openness, availability and accessibility	Author's own definition
Deficit of customer awareness	Minimum usage of sustainable and renewable material reduces material handling and transportation cost. The industry is responsible for providing waste collection and other processing facilities which if not handled carefully can deteriorate environment conditions	Ahsan and Rahman (2017), Sharma et al., (2021)

RETRACTED ARTICLE

Table 1 (continued)

Barriers	Definition	Source
Lack of green initiatives	Minimum usage of sustainable and renewable material reduces material handling and transportation cost. The industry is responsible for providing waste collection and other processing facilities which if not handled carefully can deteriorate environment conditions	Ahsan et al. (2017)
Lack of senior management dedication	Lack of involvement and encouragement from top managers, to effectively move towards Industry 4.0 and sustainability resourcefulness	Sharma et al. (20[illegible]a)
Lack of supplier support	Rigidness of suppliers and inhibitions in sharing beneficial suggestions and innovative ideas can hamper the implementation of Indus[illegible] 4.0	K[illegible]ble et a[illegible] (2018a, 2018b)

RETRACTED ARTICLE

5.2 Stage 2—quantitative (multi-crite[illegible]a de[illegible]sio[illegible] making) analysis

The research has used an integrated resea[illegible] methodology using AHP-CODAS-DEMATEL to answer the three research que[illegible]

5.2.1 RQ2: Which barriers [illegible]e criti[illegible]al to I4.0 in Indian pharmaceutical sector?

With the help of face-to-[illegible] discussions followed by AHP, this work provides classifications of the barriers (R[illegible]fer Ta[illegible]le 3) hindering I4.0 adoption. “Costly Venture”, “Deficit customer awareness”, “[illegible]ack [illegible]f sen[illegible]or management support”, “Lack of supplier flexibility”, and “Standardisatio[illegible], reg[illegible]tion, legislation and certification issues” are the top five critical barriers.

5.2.2 RQ3: W[illegible]ich potential solutions are critical for I4.0 in Indian pharmaceutical [illegible]sect[illegible]r?

[illegible]effective PSs for I4.0 adoption has been proposed using interviews with the industry [illegible]rofessionals (Refer Table 2). CODAS has been used to rank the identified potential solution. The CODAS method is a novel, effective and competent method proposed by Keshavarz Ghorabaee et al.. Recent literature has verified that this method is very beneficial and help in solving decision-making problems in uncertain environments. CODAS provide better results for innovation adoption or implementation related decision-making especially in the field of transportation, supply chain management as well as technology domain (Biswas et al., 2021; Ghorabaee & Amiri, 2017). “Customer awareness and satisfaction”, “Educating customers for recycling practices”, “Organisational/institutional awareness”, “Working on reduction of drug wastage and better drug disposal”, and “Environmental product design and life cycle

Table 2 Potential Solution proposed that may assist firms while making a decision for I4.0 adoption

Potential solution (PS)	Definition	Source
Savings (reduction in energy consumption, overproduction)	The coordinated interaction across firm and predictions using big data analytics enhances process monitoring performances thereby facilitating timely identification of errors and reduce energy consumption, overproduction, and material wastage	Ding et al. (2018)
Using 6 R (recognise, reconsider, realise, reduce, reuse/repair, recycle)	First to recognize the opportunities offered by Industrial Upcycling. Next is to evaluate and reconsider or redesign manufacturing processes for benefits business. After recognition of the opportunities and reconsideration of business processes, we need to realize the business process improvement or innovation. Next step is to reduce the use of resources to achieve efficient outcomes is the essence of the methodology. Reusing the materials considered as useable prior to process improvement is next step of progression. Finally, recycling as much as possible is one of main expected outcomes of the Industrial Upcycling effort. The aim is the zero waste	Yadav et al. (2020)
Implementation of green initiatives and sustainable practices	Manufacturing units need to reduce solid wand effluent waste, emission, as well as the capacity to cut on the consumption of harmful/toxic materials. The firms should encourage the use of preservatives as per defined standards. This provides multifold benefits like improves operational performance, increases market share, positive brand image and give a competitive edge	Sharma et al., (2021)

RETRACTED ARTICLE

RETRACTED ARTICLE

Table 2 (continued)

Potential solution (PS)	Definition	Source
Support, motivation, and job security from management	Clear conversation and right expectation from management should be discussed with employees. The managers should communicate in a manner that personnel feel motivated towards new innovations and have no fear related to job insecurities	Hofmann et al. (2017), Sharma et al., (2020b, 2020c)
Integration of digital and physical units (Transparency, Interoperability, Virtualisation)	Modular product design reduces information asymmetry and improve SC transparency. Application of digital technologies help to achieve interoperability and virtualization	Hofmann et al., (201[illegible]), Xu [illegible]al. (2018), Horváth [illegible]t al. (2019[illegible], Zhang et al. (201[illegible]
Environmental product design and life cycle analysis	Designing of product considering environmental aspects and effective life cycle analysis smoothens SSCM adoption	[illegible]iao [illegible]t al., (2017), Yadav et al., [illegible]020)
Sustainable resource management	Judicious usage of resources to reduce overuse and wastage	Zhong et al. (2017), Fatorachian and Kazemi (2018)
Adoption of safety standards	Existence of safe[illegible] stand[illegible]ds in the system ensu[illegible] empl[illegible]yee safety from acciden[illegible] during supply [illegible]	
Educating users for recycling practices	Reg[illegible]ar educa[illegible] for customers [illegible]o e[illegible]ute recycling practices assist i[illegible]mproving sustainability adoption	Batista et al. (2018), Tseng et al. (2019)
Customer awareness and satisfaction	Awareness regarding ecological, ethical, and social concerns. Customisation and personalisation with affordable prices leads to satisfied consumers	Ding et al. (2018), Kumari et al. (2018)
Working o[illegible]eduction of drug wastage and [illegible]etter drug disposal	Formulation of effective rules, strict regulations, policies for well-defined protocol for disposal of waste/drug	Ding et al. (2018)
[illegible]ustain[illegible]le production that increases customer loyalty and brand image	Sustainable practices and I4.0 increase market share (economic status) of firm, improves brand image, provides the competitive edge, reduces environmental degradation and improves the overall supply chains performance	de Sousa Jabbour et al. (2018)

Table 2 (continued)

Potential solution (PS)	Definition	Source
Policies with strict regulations	Firms should do product life assessment and follow government policies for better and sustainable products	Author own definition
Reduce GHG emissions, energy use, water wastage	With respect to the global environmental problems firms should develop eco-friendly and sustainable process to reduce GHG emissions, energy use, and water wastage	Author own definition
Organisational/institutional awareness	Working on initiatives that make other organizations recognize the long-term benefits of I4.0	Author own definition
Supplier support	Proactive interest and assistance from supplier and third -party partners towards I4.0	Author own definition
Ensuring regulatory compliance	In addition to government scrutiny the firms should follow regulatory pathways to sustain or accelerate growth rate using disruptive catalysts i.e. I4.0 technologies	[illegible] et al. (2020)

analysis" are ranked as the top five potential solution for easy implementation of I4.0. Further, "Support, motivation, and job security from management", "Educating customers for recycling practices", "Organisational and institutional awareness", and "Working on reduction of drug wastage and better drug disposal" are potential solutions that falls in cause group (Refer Table 4).

5.2.3 RQ4: What are the cause-and-effect relation among the barriers and potential solutions respectively?

In the present research, the DEMATEL is used to investigate mutual or cause-effect relationships and interdependencies as well provide causal diagram (Refer Fig. 1a and b). "Deficit customer awareness", "Costly Venture", "Standardisation, Regulations, legalisation and certification issues", "Lack of senior management support" and "Employment disruption (Skill [illegible] need for additional trainings)" are regulatory barriers that have causal influence on other barriers (Refer Table 4).

6 Discussions of findings

It is evident from the literature that specifically in emerging economies the manufacturing practices should focus not only on economic viability for better productivity but also sustainable aspects and long term societal values which is inline with our present findings (Manavalan & Jayakrishna, 2019; Sharma et al., 2021). The present work clearly explains

RETRACTED ARTICLE

Table 3 Barriers ranking using AHP and Solutions ranking using CODAS

Barriers	E	MM	SM	GM	Rank (Barriers)	Rank (S)	T (i)	E (i)	PS
I1	0.056	0.072	0.081	0.072	7	13	0.617	0.213	S1
I2	0.013	0.023	0.020	0.023	16	11	0.703	0.234	S2
I3	0.135	0.085	0.083	0.085	5	10	0.517	0.235	S3
I4	0.040	0.042	0.053	0.042	9	14	0.587	0.207	S4
I5	0.055	0.040	0.037	0.04	10	4	0.714	0.301	S5
I6	0.039	0.080	0.045	0.08	6	9	0.643	0.236	S6
I7	0.029	0.026	0.028	0.026	14	17	0.420	0.150	S7
I8	0.051	0.025	0.026	0.025	15	16	0.415	[illegible]	[illegible]
I9	0.027	0.052	0.049	0.052	8	12	0.454	[illegible]	S9
I10	0.017	0.037	0.050	0.037	11	7	[illegible]	[illegible]	S10
I11	0.022	0.033	0.033	0.033	12	2	[illegible]	[illegible]	S11
I12	0.015	0.028	0.027	0.028	13	15	[illegible]	0.198	S12
I13	0.104	0.104	0.097	0.104	2	3	[illegible]	0.322	S13
I14	0.194	0.160	0.159	0.16	1	5	[illegible]	0.290	S14
I15	0.096	0.104	0.110	0.104	3	8	0.633	0.238	S15
I16	0.107	0.089	0.102	0.089	4	[illegible]	0.787	0.290	S16
						[illegible]	0.786	0.351	S17

I1, Employment disruption (Skill gaps and need for additional trainings etc.); I2, Counterfeit and absence of correct information; I3, Standardisation, regulations, localisation and certification issues; I4, Poor openness, accessibility and availability; I5, Lack of simple and fair benchmarking; I6, Infrastructure standardisation; I7, Disproportion between technology development and social evolution; I8, Automation and digitisation of process; I9, Value chain integration's challenges; I10, Ineffectiveness in change management; I11, Automation error; I12, Lack of green initiatives; I13, Deficit of customer awareness; I14, Costly Venture; I15, Lack of senior management support; I16, Lack of supplier flexibility

SM, Senior Management; MM, Middle Management; E, Executives; GM, Geometric Mean

PS, Potential Solution; S1, Sustainable Resource Management; S2, Integration of Digital and Physical Units (Transparency, Interoperability, Visualisation); S3, Support, motivation, and job security from management; S4, Implementation of Green Initiatives and sustainable practices; S5, Working on reduction of drug wastage and better drug disposal; S6, Savings (Reduction in Overproduction, Energy consumption, material wastage); S7, Adoption of safe standards; S8, Reduce GHG emissions, energy use, water wastage; S9, Using 6 R (recognise, reconsider, realise, reduce, reuse/repair, recycle); S10, Ensuring Regulatory Compliance; S11, Educating customers for recycling practices; S12, Supplier Support; S13, Organisational/institutional awareness; S14, Environmental Product Design and life cycle analysis; S15, Policies with strict regulations; S16, Sustainable production that increases Customer loyalty and brand image; S17, Customer awareness and satisfaction

R(i), Euclidean distances; T(i), Taxicab distances

RETRACTED ARTICLE

prisoners' dilemma which states that decision makers should emphasize on both present as well as future needs hence focus on sustainability while making technology adoption decision. If a firm want to excel it should not wait for the hundredth monkey effect rather should be one of the front runner and exploit full market potential and capture most of its share and enjoy the competitive advantage in future. The primary decision to migrate towards any innovation is governed by the economic value that innovation provides (Stentoft et al., 2021). It's difficult to find firms that are willing to invest money on emerging technologies since they are not sure if they will be able to monetise from them as there is no assured definite expected outcome. For a firm to adopt any technology it is essential that it has sufficient funds to work towards its implementation. If the initial investment is too high, the firm delay

RETRACTED ARTICLE

Table 4 DEMATEL Analysis: Cause -Effect Relatio[illegible]ps in Barriers and Potential Solutions

BR	R	D	D + R	R–D	Rk (BR)	CE (BR)	CE (SI)	Rk (SI)	R–D	D + R	D	R	PSs
B1	8.831	8.520	17.351	0.310	[illegible]	C	C	3	0.06	0.90	0.42	0.48	S1
B2	7.901	8.483	16.385	– 0.582	9	E	E	5	– 0.05	1.07	0.56	0.51	S2
B3	8.116	8.737	16.853	– 0.622	10	E	C	4	0.04	1.15	0.56	0.59	S3
B4	6.430	6.779	13.210	– 0.349	8	[illegible]	E	8	– 0.17	0.90	0.53	0.37	S4
B5	9.012	8.129	17.141	0.883	1	C	E	9	– 0.13	0.86	0.49	0.36	S5
B6	7.893	8.171	16.063	– 0.278	7	E	E	10	– 0.31	0.67	0.49	0.18	S6
B7	7.882	7.636	15.518	0.246	5	C	C	2	0.29	1.05	0.38	0.67	S7
B8	7.995	7.656	15.651	0.340	2	C	C	1	0.40	1.10	0.35	0.75	S8
B9	7.690	7.372	15.062	0.318	3	C	E	6	– 0.06	0.75	0.40	0.34	S9
B10	6.748	7.013	13.761	– 0.265	6	E	E	7	– 0.07	0.50	0.28	0.22	S10

BR: Barriers; B1, Lack of senior management support; B2, Value chain integration's challenges; B3, I[illegible]astructure standardisation; B4, Lack of simple and fair benchmarking; B5, Deficit customer awareness; B6, Poor openness, accessibility and availability; B7, Employment [illegible]ruptio[illegible]Skill Gaps, Need for additional trainings); B8, Costly Venture; B9, Standardisation, regulations, legalisation and certification issues; B10, Lack of supplier flexibility

PS, Potential Solution; S1,Organisational and institutional awareness; S2, Environmental Product Design [illegible] [illegible]e cycl[illegible] analysis; S3, Working on reduction of drug wastage and better drug disposal; S4, Policies with strict regulations; S5, Savings (Reduction in Overproduction, Energy [illegible]nsumpt[illegible] material wastage); S6, Support, motivation, and job security from management; S7, Educating customers for recycling practices; S8,Customer awareness and satisfaction[illegible], S[illegible]tainable production that increases Customer loyalty and brand image; S10, Ensuring regulatory compliance; R, Sum of Rows; D, Sum of Columns; Rk, Rank; CE, Cau[illegible]-effect[illegible] Cause; E, Effect

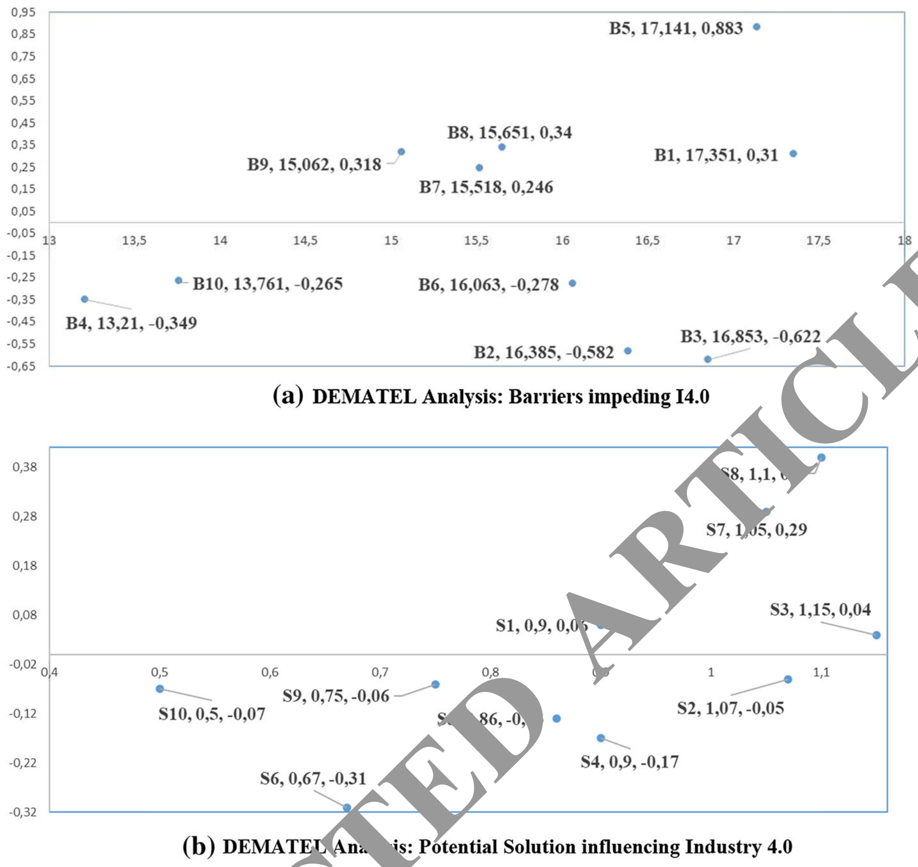

RETRACTED ARTICLE

Fig. 1 DEMATEL Analysis: Barr[illegible]rs (1a) [illegible]nd Potential Solution (1b) influencing Industry 4.0 **a** DEMATEL Analysis: Barriers impeding I4.0. [illegible]: Lack of green initiatives; B2: Lack of senior management support; B3: Costly Venture; B4: St[illegible]rdisation, Regulations, Legalisation and Certification issues; B5: Employment Disruption (Skill Gaps, Need [illegible]ditional training); B6: Lack of supplier flexibility; B7: Lack of simple and fair benchmarking; [illegible] Poor [illegible]penness, accessibility and availability; B9: Value chain Integration's challenges; B10: Infrastruct[illegible] St[illegible]ndardization
b DEMATE[illegible] Ana[illegible]is: [illegible]otential Solution influencing Industry 4.0. *Note* S1: Environmental product design and life c[illegible] analysi[illegible] S2: Policies with strict regulation; S3: Savings (Reduction in Overproduction, Energy consump[illegible]tion, [illegible]aterial wastage), S4: Ensuring regulatory compliance; S5: Support, motivation, and job security fro[illegible] manageme[illegible]t; S6: Working on reduction of drug wastage and better drug disposal; S7: Sustainable p[illegible]n that increases Customer loyalty and brand image; S8: Organizational and institutional awareness; S9: E[illegible]cating customers for recycling practices; S10: Customer awareness and satisfaction

the decision of adoption owing to lack of funds or blocking the funds in a single venture. Firms want to meet the dynamic needs but experimenting with collaborative working and intelligent manufacturing needs prior researched cases. Innovator firms are the first to explore any technology and other follows their path if their risks lead to successful ventures. This is in line with our findings that emphasize that "costly venture" is the main impediment when a firm think of implementing I4.0t (Nimawat & Gidwani, 2021). I4.0t helps a firm to work on simulated models and has the opportunity to correct the errors before they encounter them in real scenario. Hence, a firm can perform rapid experimentation, prototyping and

faster concept testing. This saves a lot of cost which needs to be emphasised upon when a firm make a decision of investment. I4.0t allows continuous cloud-based energy monitoring that helps in successful execution of energy management thereby reducing cost related to unwanted energy wastage. Hence the potential solution i.e., "savings (Reduction in energy consumption and overproduction,)" should be discussed with potential adopters which can motivate firms to move towards I4.0t and promote sustainability practices.

The other critical issue that needs immediate attention is "Deficit customer awareness". Customers do not have sufficient knowledge on how to minimise wastage. There are practices that emphasize on recycle practices but minimisation of waste production needs immediate attention. The customers should be committed with application of sustainable practices so that the present activities does not cause any adverse effect on future. There should be seminars, webinars, and conferences for consumer awareness. Government should advertise, use social media platforms, and have dedicated programs ensuring the end-customer have sufficient knowledge. The present findings also reveal that the most critical potential solutions are" customer awareness and satisfaction" and "educating customers for recycling practices".

"Lack of senior management support" acts as a primary inhibitor [illegible] I4.0t implementation in a firm. Firms need to enhance strategic organisational policies, personnel expertise, improved governance, and employee-friendly professional practices. Proactive support from top management is essential for the smooth implementation of any innovation (Sharma et al., 2020a, 2020c). Hence, firms should emphasis on a more dedicated methodology to manage organisational challenges (Teodosiu & Castells, 2017). A firm should help employees to look for a wider picture and look for long term benefits. If the employee feels motivated to accept new changes and upgrade his/her skills by his/her own will then the innovation is better accepted and have chances of better outcomes.

"Lack of supplier flexibility" is another important deterrent. Non-cooperating supplier can impede firms from moving towards new technological paradigm (de Sousa Jabbour et al., 2018; Horváth et al., 2019). The firms should have a good understanding and support from suppliers. It is critical since their flexibility and network collaboration are also of prime importance while making any new technology related decision.

"Lack of Standardisation, Regulations, Legalisation and Forms of Certification" and "" are yet another impediment when a firm makes a decision towards technology advancements. I4.0 global standards, government policies and information sharing procedures are required for developing smart production systems (de Sousa Jabbour et al., 2018). Müller et al. (2018) recommended that firms need global standardisation, governmental support for eliminating I4.0 challenges in initial stages. Well defined protocols and policies can aid the manufacturing firms to convert traditional industrial units to industrial units of the future.

"Employment Disruption (skill gaps, need for additional trainings)" is another major blocker against I4.0 implementation. It is critical to understand that robots are only programmable machine that can implement tedious tasks and act as idyllic human confidante in repetitive tasks. This makes it clear that robots will support human in jobs that do not call for a real-time decision-making and will not cause any employment disruption (Nahavandi, 2019). Firms need to support its employees in understanding the benefits of digitalisation rather than concentrating on fabricated dystopian view.

"Working on reduction of drug wastage and better disposal", "Environmental product design and life cycle analysis" and "Sustainable production that increases customer loyalty and brand image" are the three critical potential solutions to motivate firms to move towards I4.0. A small initiative can be minimising medications wastage that will reduce large scale dumping of expired or unwanted medicines. Any firm that concentrates on greening their

RETRACTED ARTICLE

supply chain or green procurement processes are expected to have better business performance in coming future. Especially after COVID-19 the end customers are willing to pay more and looking for brands that provide sustainable and greener solutions (Sharma et al., 2023b). These potential solutions will help in increasing firm's brand value among end users and eventually an increase in their market share (de Sousa Jabbour et al., 2018; Kazancoglu and Ozkan-Ozen, 2018).

I4.0 is expected to be a highly revolutionary technology where production of small batches of drugs, each with custom dosages, shapes, sizes and release characteristics is possible. I4.0 helps in the manufacturing of personalized medicines where the focus is on using principles that guides them to judiciously use the available resources. I4.0 facilitates the creation of sustainable value, leads to a more agile, intelligent and personalized pharmaceutical industry and, in the long run, allows pharmaceutical companies to obtain competitive advantages (Sharma et al., 2023a). A firm need to take difficult decision in situations that are complex, so a firm should follow more formal and systematic approach to decision-making. Hence, a firm should set clear goals and set definite objectives in order to achieve better results. Presently, firms need solutions where they can function effectively in man–machine collaboration using advanced techniques like sensors, audio and human intention analysis. One easy solution is Collaborative Robots (CoRobs) that allows flexible and sound working environment. Based on the scanning and interpretation of the product's information from beacons, sensors and tags, the CoRobs can determine when and where it needs to pass information and products such as in deciding for wafers' time and location of batch placement (Ivanov et al., 2019). AI backed big data analytics empowers firms with real-time decision-making to gain competitive advantage (Chen et al., 2012). The principal objective of I4.0+ is to solve social problem and stabilise financial progression which can be only be achieved by using a collaboration that mimics the signals, constructions and processes (refer Fig. 2).

RETRACTED ARTICLE

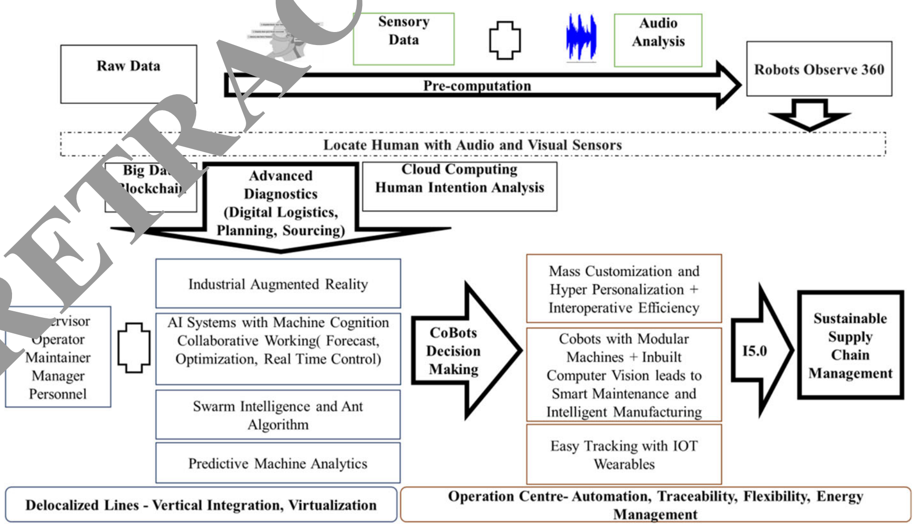

Fig. 2 Working with Industry 4.0 technology. Alt Text: Describes how Industry 4.0 functions and role of human–machine interaction leading towards Industry 5.0 for sustainable supply chain management

The aim is to develop an advanced-diagnostics having near-zero error or failure rate for sustainable, mass customised, superior quality manufacturing with human-centered management (Sharma et al., 2022a). CoRobs play a very critical role in SCM. Routine tasks including quality checks, packaging, heavy good transportation can be done by robots, and human can use his expertise in more complex or advanced tasks. CoRobs can easily streamline SCM processes by easily repeating mechanical tasks such as material assembly, packing, quality checks, transportation, product delivery to the consumers and pick-ups of product return from the consumers thereby also reducing total ownership cost. CoRobs enhance dexterity and can easily perform dangerous, repetitious or physically challenging tasks. CoRobs work in harmony with human, thereby using his expertise to produce personalized and mass customized products with high speed and accuracy.

Also, advanced diagnostics such as augmented reality, artificial intelligence with machine cognition allows interoperability, better customization, network intelligence and real time forecasting. Technologies like blockchain and big data allows decentralized management, better decision making, operational transparency with reduced costs.

Internet of things acts a boon for manufacturing firms where firms can easily analyze by monitoring the digital version of final products or processes. This can avoid total failures and unexpected problems that may occur while processing a batch in the real world. The amalgamation of big data, artificial intelligence, and machine learning help in reducing defects, inventory and maintenance costs and enhance overall system performance. These technologies make best use of data thereby diagnosing and eradicating irrelevant practices/approaches and enhancing predictability and exploring innovative opportunities. Using the interconnection of multiple factory data these advanced technologies enable intelligent manufacturing thereby producing mass customizable products. Hence, we propose that "*collaborative and cognitive working'* will lead to smart manufacturing that helps a firm to have SSCM.

RETRACTED ARTICLE

6.1 Implications of the research

The research offers two important academics and four practical implications to academicians, senior management and government bodies.

6.1.1 Academic implications

First, the present work has followed a detailed and sequential mixed- methodology approach to unearth the barriers and propose potential solutions for the impediments that barricade the adoption of I4.0. This is the first study that has integrated AHP-CODAS-DEMATEL techniques. The output from AHP acts as an input to CODAS; i.e., integration of these [illegible] methods leads to synchronised solution. The relative weights from AHP has been used to find which potential solution are of utmost importance in present context. Further, the identification of accurate and critical barriers is of utmost importance owing to two reasons a) to understand if these barriers are actually impacting the decision making and identifying the underlying causes; b) to look for means and solutions that can help in removing such impediments. The identification of criticality is also very important since the barriers priority can be different in different firms. It is also seen that sometimes the firm delay critical decisions thinking if it is well conceived by the employees however examining the viewpoint of employees can only express if the employees are rigid or are they open to changes. Hence, the factor may be accurate but is not critical for a firm where employees are open to innovations.

The detailed methodology is very critical because it helps to identify criticality as well as cause-effect groups. The empirical evidence gives a wider picture by classifying which factors fall under influencing (cause) group and which factors under influenced (effect) group. For a decision-making firm it is recommended that they first cater cause group factors since they not only impact the system themselves but also influence other factors which may change the holistic landscape of decision making.

Second, the proposed framework will help firms to understand the functioning and improvements that I4.0 will bring to the firm. This framework articulates the role of investment, standardisation and technology on I4.0 adoption intention. Moreover, our research uncovered important relationships between human–machine interaction which is key to success for coming generations especially in manufacturing. Human–machine interaction depends on safety, reliability and standardisation which are discussed theoretically but needs immediate attention in terms of implementation. It is also important that there should be well-articulated and well-defined regulations that can provide assurance to all the stakeholders. For example, firms feel insecure if technologies like big data, cloud computing and IoT are implemented as then they have a misconception that their data is at stake. Technology is the undeniable truth for future. As a firm that wants to grow, it is needed that they work in direction of advancements and accept technology as a partner in their business. However, adopting anything just because others are implementing is not a right approach. A firm should clearly understand their requirements and thoroughly check the regulations and policies related to the technologies. For example, if the data is sensitive then adopting a private cloud is most advisable option. However, if the firm has limited funds, then it is important to segregate data into very critical, critical, less critical and non-critical. In such conditions a firm can adopt hybrid clouds where more critical data can be stored on private cloud and less critical and non-critical on public cloud.

Third, the findings contribute to the body of knowledge by giving a clear picture of which impediments need to be overcome and how a firm can achieve this. The prior literature has given disproportionate weight to solution identification as most of the work identify the problem and rank the barriers. Hence, this is one of the few works that provide clear and holistic understanding to both academicians as well as practitioners.

6.1.2 Practical implications

Our findings propose that the transparent and real-time information sharing with I4.0-advanced diagnostics motivate more effective collaboration and communication among manufacturers, distributors, suppliers, hospitals and end-consumers. The framework is very critical since it gives a clear picture to all stakeholders about the functioning and benefits of I4.0. A manufacturing firm spends and loses a lot of money if the defects are found while the firm is actually operating in the product lifecycle. It is observed that IoT and AI provides the capability of detecting counterfeit products early in the product life cycle by working on prototypes and data modelling. These technologies save a lot of time, money and energy. At the same time, it allows faster and effective modelling and prototype testing which can revolutionise the industry as a whole. Disruptive technologies require increased regulatory checks so that the critical data is not compromised. Hence, there is a need that firms remain updated with the regulations and certifications.

One of the biggest issues with pharmaceutical manufacturing is rising consumer expectations and difficulties managing brand health. The customised solutions with I4.0t can help the manufactures to have an edge over other competitors and have first mover advantage by quickly capturing the market. Sometimes customisation is not an added advantage as one size

RETRACTED ARTICLE

does not fits all. Smart manufactures are using technologies like AI, BDA for making mass customisation possible in order to boost customer satisfaction that may win brand loyalty in long run. These innovative manufacturing firms use digital capabilities and transforming the pharmaceutical manufacturing business.

Second, human intention analysis and apt usage of human expertise help in early detection of potential risks that may lead to system failure. This help in on-time execution of right strategies that will reduce wastage and leads to better utilisation of resources thereby ensuring sustainable supply chain management (Sharma et al., 2022b).

Third, we propose that stakeholders can use connected systems and move towards real-time monitoring for an agile and resilient manufacturing unit. The main benefit for such a simulated environment is that automatic self-adjustments can be done in processes as per the data trends and information processed. Since data is the soul and heart of I4.0 and data analysis techniques such as AI, ML and DL enable simulations through which the system can learn and optimize product quality.

Fourth, since inception of Industry 4.0, it is seen as the driver that enhances efficiency and effectiveness of production processes thereby act as the enabling element of manufacturing firms which meliorates "Smart Factory". However, present research emphasis that I4.0 is actually a paradigm shift where focus is moving from the individual plants to a comprehensive visualisation of the whole supply chain. Hence, I4.0 is amalgamation of digitalisation as well as the concept of sustainability & circular economy. I4.0 enables the smart supply chain that focusses on the circular economy.

Fifth, we recommend the "Educating customers for recycling practices" should be of utmost importance for coming years. Indian consumers are brand conscious since a large section follow influencers and actors without any questioning and sometimes even blindly. Hence, brand ambassadors have an important role to play in influencing consumer behaviour especially in Indian market (Goutam, 201[illegible]). Though there have also been empirical evidences that indicate that normative values, social influence, and group esteem affect consumers' intention to move towards green initiatives (Khare et al., 2013). Hence, we propose the government should take initiatives and our leaders should address issues related to recycling practices and green initiatives. Self-driven customers and social influences from peer groups can influence other consumers with the help of right branding and awareness campaigns.

Sixth, we propose that 'Customer awareness and satisfaction' is the backbone of success in this ever-evolving environment of pharmaceutical production. It is the duty of government and big players in the market to arrange webinars, seminars, conferences to generate awareness in the consumers. As a developing country with in numerous manpower strength, we should understand that concerns related to ecological, ethical, and social issues need immediate attention. Firms try to cost optimise their solutions but if planet concerns are the central issues, then customers should also be willing to understand and pay for environmentally solutions.

Seventh, real-time data monitoring helps in keeping a close eye on the resources consumption and responds to production management. Moreover, digitalisation will help factories by reducing the use of materials with the adoption of additive manufacturing (3D printing) thereby less wasteful of resources than conventional subtractive methods.

RETRACTED ARTICLE

7 Conclusions, limitations, and scope for future research

This research has investigated barriers, suggested potential solutions, that may assist potential adopters to move towards I4.0. The work also categorises the barriers and potential solutions in cause-and-effect group thereby giving a clear understanding to potential adopters which factors need more attention while making an adoption decision. The work emphasizes that firms should look for holistic picture and beyond investment cost. We also make a proposition that customer and organisational awareness can accelerate understanding of how emerging technology can lead to sustainable solutions. The work tries to explore and finally revolves around 5 important research questions (**RQ**). In order to answer the **first** and **third RQ** respectively, author have done extensive literature review followed by detailed interviews to investigate the comprehensive list of barriers and propose potential solutions. Further to answer **RQ2**, AHP analysis is conducted which clearly highlights that "Costly Venture" & "Deficit of customer awareness" are the critical impediments. The CODAS analysis proposes that "Organisational/institutional awareness" & "Environmental Product Design and life cycle analysis" as the critical potential solutions. Finally, in response to **RQ4 and RQ5** DEMATEL analysis is utilised which proposes that "Deficit customer awareness" is the most critical causal barrier while "Customer awareness and satisfaction" is the most critical causal solution in response to the identified barrier which demands immediate attention from all stakeholders. Further, this is the first work that proposes a human–machine interaction framework leading towards Industry 5.0 for sustainable supply chain management. This framework will pave path for future researchers and practitioners to understand the importance of human-centricity, sustainability, and resiliency. The contributions of this research should be seen in the light of its limitations. First, the authors have used expert opinions from India that limits the generalisability of results in other geographies that are advanced and more developed. Even in other developing countries the scenario is different, one such example is China. China lacks manufacturing technology though it is continuously working vigorously in field of science and technology but has imperfect standard system of industrialization (Feng et al., 2018). Hence, our findings may not resonate with such cases. Second, the results are based on knowledge of 14 experts. Hence, the author propose that more cross sectional and longitudinal works can be conducted. We also propose future researchers to use optimisation and machine learning approaches to simulate the findings thereby predicting and proposing better solution. Third, within the same sector findings may vary; i.e., the pharmaceutical manufacturing sector is very different form electronics or automotive sectors. Hence similar works or cross-sectional studies should be done in other sectors to identify relevant barriers in order to accelerate I4.0 adoption. Fourth, the this work has presented the barriers and solutions by categorising under different dimensions; however, future works can use a detailed theoretical underpinning and can test the same using survey method. The future scholars can explore behaviour reasoning theory, theory of reasoned action or other relevant frameworks to investigate the phenomenon under question. The future studies can also compare different MCDM techniques and provide comparative results on the same theme either on single country or on different countries. Also, the present work only proposes a framework, future scholars can empirically test the relevance of same using multiple case studies. Finally, more recently, generative AI-based tools and applications, such as ChatGPT, BARD (Dwivedi et al., 2023a), and technologies like metaverses (Dwivedi et al., 2022, 2023b), have emerged. They are projected to play an important role in supporting various activities across different industries. Future studies should also examine the role of these nascent technologies and applications in the context of Industry 4.0.

RETRACTED ARTICLE

Funding This study was not funded by any institute or organisation.

Data availability The data that support the findings of this study are available from the first author, [MS], upon reasonable request.

Declarations

Conflict of interest Mahak Sharma declares that she has no conflict of interest. Rajat Sehrawat declares that he has no conflict of interest. Mihalis Giannakis declares that he has no conflict of interest. Yogesh K Dwivedi declares that he has no conflict of interest.

Ethical approval This article does not contain any studies with animals performed by any of the authors.

Informed consent Informed consent was obtained from all individual participants included in the study.

Open Access This article is licensed under a Creative Commons Attribution 4.0 International License, which permits use, sharing, adaptation, distribution and reproduction in any medium or format, as long as you give appropriate credit to the original author(s) and the source, provide a link to the Creative Commons licence, and indicate if changes were made. The images or other third party material in this article are included in the article's Creative Commons licence, unless indicated otherwise in a credit line to the material. If material is not included in the article's Creative Commons licence and your intended use is not permitted by statutory regulation or exceeds the permitted use, you will need to obtain permission directly from the copyright holder. To view a copy of this licence, visit http://creativecommons.org/licenses/by/4.0/.

Appendix A

See Table 5.

Table 5 Profile of Respondents

Participant	Age	Qualification	Experience (Years)	Gender	Position
Firm A					
1	57	Post-Graduate	35	Male	Managing Director
2	56	Post-Graduate	33	Male	Director (Tech)
3	48	Doctorate	16	Female	Senior Research Analyst
4	44	Post-Graduate	20	Male	Senior Business Analyst
5	[illegible]4	Post-Graduate	18	Male	Project Manager
6	42	Post-Graduate	16	Female	Commercial Analyst
Firm B					
[illegible]	45	Graduate	23	Male	Managing Director and Owner
8	43	Graduate	10	Female	President and Co-Owner
9	40	Post Graduate	18	Male	Operations Head
10	38	Doctorate	10	Male	Research Head
11	37	Graduate	15	Male	Warehouse Head
12	35	Graduate	13	Male	Logistics Senior Manager
13	33	Graduate	12	Female	Logistics Manager

RETRACTED ARTICLE

Table 5 (continued)

Participant	Age	Qualification	Experience (Years)	Gender	Position
14	31	Graduate	9	Female	Business Analyst
15	29	Post-Graduate	4	Female	Marketing and Sales Manager
16	27	Graduate	6	Female	Production Manager
17	27	Post-Graduate	4	Male	Senior Research Analys[illegible]
18	25	Post-Graduate	2	Female	Research Analyst
19	25	Graduate	4	Male	Transportation [illegible]xecutive
20	25	Graduate	4	Male	Logistics Ex[illegible]cuti[illegible]

RETRACTED ARTICLE

References

Ahsan, K., & Rahman, S. (2017). Green public procurement implementation [illegible]allenges in Australian public healthcare sector. *Journal of Cleaner Production, 152*, 181–19[illegible]

Ancarani, A., & Mauro, C. D. (2018). Reshoring and Industry 4.0[illegible] h[illegible]ften do they go together? *IEEE Engineering Management Review, 46*(2), 87–96.

Arden, N. S., Fisher, A. C., Tyner, K., Yu Lawrence, X., [illegible] S. L., & Kopcha, M. (2021). Industry 4.0 for pharmaceutical manufacturing: preparing for the smart [illegible]ctories of the future. *International Journal of Pharmaceutics, 602*, 120554.

Aslam, F., Aimin, W., Li, M., & Rehman, K. U. (2020). In[illegible]ation in the era of IoT and industry 5.0: Absolute innovation management (AIM) framew[illegible] *I[illegible]ormation, 11*(2), 124.

Bag, S., Gupta, S., & Kumar, S. (2021). [illegible]ndustry [illegible]0 adoption and 10R advance manufacturing capabilities for sustainable development. *Inter[illegible]nal Jou[illegible]nal of Production Economics, 231*, 107844.

Bai, C., Dallasega, P., Orzes, G., & [illegible]rkis, [illegible]2020). Industry 4.0 technologies assessment: A sustainability perspective. *International J[illegible]urnal of Pro[illegible]uction Economics, 229*, 107776.

Basl, J. (2017). Pilot study of re[illegible]diness [illegible]f Czech companies to implement the principles of Industry 4.0. *Management and Production [illegible]ineering Review, 8*(2), 3–8. https://doi.org/10.1515/mper-2017-0012

Batista, L., Bourlakis, M., [illegible]art, P., & Maull, R. (2018). In search of a circular supply chain archetype–a content-analysis-base[illegible] [illegible]ure review. *Production Planning & Control, 29*(6), 438–451.

Bhatia, M. S., & [illegible]ar, S. (2020). Critical success factors of industry 4.0 in automotive manufacturing industry. *I[illegible]E [illegible]ansactions on Engineering Management, 69*(5), 2439–2453. https://doi.org/10.1109/TEM.2[illegible]0.3[illegible]7004

Biswas, S., [illegible]amucar, [illegible]., Kar, S., & Sana, S. S. (2021). A new integrated FUCOM–CODAS framework with Fe[illegible]mate[illegible] fuzzy information for multi-criteria group decision-making. *Symmetry, 13*(12), 2430.

Bog[illegible]viz, A. V., [illegible]sipov, V. S., Chistyakova, M. K., & Borisov, M. Y. (2019). Comparative analysis of formation o[illegible] indu[illegible]stry 4.0 in developed and developing countries. *Industry 4.0: industrial revolution of the 21st [illegible]ntury* (pp. 155–164). Springer.

[illegible]hen, [illegible]., Chiang, R. H. L., & Storey, V. C. (2012). Business intelligence and analytics: From big data to big impact. *MIS Quarterly* , 1165–1188

[illegible]alenogare, L. S., Benitez, G. B., Ayala, N. F., & Frank, A. G. (2018). The expected contribution of Industry 4.0 technologies for industrial performance. *International Journal of Production Economics, 204*, 383–394.

Ding, B. (2018). Pharma Industry 4.0: Literature review and research opportunities in sustainable pharmaceutical supply chains. *Process Safety and Environmental Protection, 119*, 115–130.

Dwivedi, Y. K., Kshetri, N., Hughes, L., Slade, E. L., Jeyaraj, A., Kar, A. K., & Wright, R. (2023a). "So what if ChatGPT wrote it?" Multidisciplinary perspectives on opportunities, challenges and implications of generative conversational AI for research, practice and policy. *International Journal of Information Management, 71*, 102642.

Dwivedi, Y. K., Hughes, L., Wang, Y., Alalwan, A. A., Ahn, S. J., Balakrishnan, J., & Wirtz, J. (2023b). Metaverse marketing: How the metaverse will shape the future of consumer research and practice. *Psychology & Marketing, 40*(4), 750–776.

Dwivedi, Y. K., Hughes, L., Baabdullah, A. M., Ribeiro-Navarrete, S., Giannakis, M., Al-Debei, M. M., & Wamba, S. F. (2022). Metaverse beyond the hype: Multidisciplinary perspectives on emerging challenges, opportunities, and agenda for research, practice and policy. *International Journal of Information Management, 66*, 102542.

Fareri, S., Fantoni, G., Chiarello, F., Coli, E., & Binda, A. (2020). Estimating Industry 4.0 impact on job profiles and skills using text mining. *Computers in Industry, 118*, 103222. https://doi.org/10.1016/j.compind.2020.103222

Fatorachian, H., & Kazemi, H. (2018). A critical investigation of Industry 4.0 in manufacturing: Theoretical operationalisation framework. *Production Planning & Control, 29*(8), 633–644. https://doi.org/10.1080/09537287.2018.1424960

Feng, L., Zhang, X., & Zhou, K. (2018). Current problems in China's manufacturing and countermeasures [illegible] industry 4.0. *EURASIP Journal on Wireless Communications and Networking, 2018*, 1–6.

Frank, A. G., Dalenogare, L. S., & Ayala, N. F. (2019). Industry 4.0 technologies: Implementation p[illegible]tterns in manufacturing companies. *International Journal of Production Economics, 210*, 15–26. https:/[illegible]i.org/10.1016/j.ijpe.2019.01.004

Gabus, A., & Fontela, E. (1973). Perceptions of the world problematique: Communication [illegible]ocedure, communicating with those bearing collective responsibility (pp. 11–18). (DEMATEL r[illegible]port [illegible] 1). [illegible]attelle Geneva Research Centre

Geissbauer, R., Schrauf, S., & Koch, V. (2014). Industry 4.0: Opportunities and challe[illegible]ges of i[illegible]strial internet, PricewaterhouseCoopers. Freudenberg IT.". Available at: https://www.pwc.n[illegible]/en/[illegible]ets/documentAhlers, T., 2015. Industrie 4.0: The Big Unknown? Freudenberg IT.

Ghobakhloo, M. (2018). The future of manufacturing industry: A strate[illegible]ic r[illegible]admap toward Industry 4.0. *Journal of Manufacturing Technology Management, 29*(6), 910–936.

Ghorabaee, M. K., Amiri, M., Zavadskas, E. K., Hooshmand, R., & Antuchevič[illegible]e, J. (2017). Fuzzy extension of the CODAS method for multi-criteria market segment evalua[illegible]ournal of Business Economics and Management, 18*(1), 1–19. https://doi.org/10.3846/16111699.2016.12[illegible]8559

Goutam, D. (2013). Influence of brand ambassadors on buying beha[illegible]ior of soft drinks: With reference to Belgaum City. *International Journal of Research i[illegible]ness Management, 1*(4), 9–18.

Hofmann, E., & Rüsch, M. (2017). Industry 4.0 and t[illegible] curren[illegible]status as well as future prospects on logistics. *Computers in Industry, 89*, 23–34.

Horváth, D., & Szabó, R. Z. S. (2019). Driving [illegible]orces and [illegible]riers of Industry 4.0: Do multinational and small and medium-sized companies have equal [illegible]ortun[illegible]ies? *Technological Forecasting and Social Change, 146*, 119–132.

Ivanov, D., Dolgui, A., & Sokolov, B. [illegible] The impact of digital technology and Industry 4.0 on the ripple effect and supply chain risk [illegible]tics. [illegible]rnational Journal of Production Research, 57*(3), 829–846. https://doi.org/10.1080/002[illegible]7543.2018.[illegible]488086

Kamble, S. S., Gunasekaran, A., [illegible] Sharma, R. (2018a). Analysis of the driving and dependence power of barriers to adopt industry 4.0 [illegible]dian manufacturing industry. *Computers in Industry, 101*, 107–119.

Kamble, S. S., Gunasekara[illegible] Gawankar, S. A. (2018b). Sustainable Industry 4.0 framework: A systematic literature review identi[illegible]ing [illegible]he current trends and future perspectives. *Process Safety and Environmental Protection, [illegible]*, [illegible]08–4[illegible]5.

Kamble, S., Gu[illegible]A., & Dhone, N. C. (2020). Industry 4.0 and lean manufacturing practices for sustainable o[illegible]nisational performance in Indian manufacturing companies. *International Journal of Pro[illegible]on Research, 58*(5), 1319–1337.

Kaza[illegible]oglu, [illegible] & Ozkan-Ozen, Y. D. (2018). Analyzing workforce 4.0 in the fourth industrial revolution and proposing a road map from operations management perspective with fuzzy DEMATEL. *Journal of [illegible]nterp[illegible]ise Information Management*. https://doi.org/10.1108/JEIM-01-2017-0015

[illegible]esha[illegible] Ghorabaee, M., Zavadskas, E. K., Turskis, Z., & Antucheviciene, J. (2016) A new combinative distance-based assessment (CODAS) method for multi-criteria decision-making." *Economic Computation & Economic Cybernetics Studies & Research* 50(3).

[illegible]hare, A., Mukerjee, S., & Goyal, T. (2013). Social influence and green marketing: An exploratory study on Indian consumers. *Journal of Customer Behaviour, 12*(4), 361–381.

Kiel, D., Arnold, C., & Voigt, K.-I. (2017). The influence of the Industrial Internet of Things on business models of established manufacturing companies–A business level perspective. *Technovation, 68*, 4–19.

Kshetri, N. (2018). 1 Blockchain's roles in meeting key supply chain management objectives. *International Journal of Information Management, 39*, 80–89. https://doi.org/10.1016/j.ijinfomgt.2017.12.005

Kumar, M., Graham, G., Hennelly, P., & Srai, J. (2016). How will smart city production systems transform supply chain design: A product-level investigation. *International Journal of Production Research, 54*(23), 7181–7192.

RETRACTED ARTICLE

Kumari, A., Tanwar, S., Tyagi, S., & Kumar, N. (2018). Fog computing for Healthcare 4.0 environment: Opportunities and challenges. *Computers & Electrical Engineering, 72*, 1–13.
Leitão, P., Colombo, A. W., & Karnouskos, S. (2016). Industrial automation based on cyber-physical systems technologies: Prototype implementations and challenges. *Computers in Industry, 81*, 11–25.
Liao, Y., Deschamps, F., de Freitas, E., Loures, R., & Ramos, L. F. P. (2017). Past, present and future of Industry 4.0-a systematic literature review and research agenda proposal. *International Journal of Production Research, 55*(12), 3609–3629.
Lopes de Sousa Jabbour, A. B., Jabbour, C. J. C., Filho, M. G., & Roubaud, D. (2018). Industry 4.0 and the circular economy: A proposed research agenda and original roadmap for sustainable operations. *Annals of Operations Research, 270*(1), 273–286.
Luthra, S., & Mangla, S. K. (2018). Evaluating challenges to Industry 4.0 initiatives for supply chain susta[illegible]ability in emerging economies. *Process Safety and Environmental Protection, 117*, 168–179.
Machado, C. G., Winroth, M. P., & da Silva, E. H. D. R. (2020). Sustainable manufacturing in Ind[illegible]stry 4.0: An emerging research agenda. *International Journal of Production Research, 58*(5), 1462–148[illegible]
Maddikunta, P. K. R., Pham, Q.-V., Prabadevi, B., Deepa, N., Dev, K., Gadekallu, T. R., Rub[illegible], R., & [illegible]an-age, M. (2022). Industry 5.0: A survey on enabling technologies and potential applica[illegible]ons. *Jour[illegible]al of Industrial Information Integration, 26*, 100257.
Makkonen, H. (2021). Information processing perspective on organisational innov[illegible]ion [illegible]doption process. *Technology Analysis & Strategic Management, 33*(6), 612–624.
Manavalan, E., & Jayakrishna, K. (2019). A review of Internet of Things (IoT) [illegible]nbe[illegible]ed sustainable supply chain for industry 4.0 requirements. *Computers & Industrial Engineerin[illegible]27*, 92[illegible]53. https://doi.org/10.1016/j.cie.2018.11.030
Mittal, S., Khan, M. A., Romero, D., & Wuest, T. (2018). A critical review o[illegible]anufacturing & Industry 4.0 maturity models: Implications for small and medium-sized enterprise[illegible]MEs). *Journal of Manufacturing Systems, 49*, 194–214. https://doi.org/10.1016/j.jmsy.20[illegible]005
Moeuf, A., Lamouri, S., Pellerin, R., Tamayo-Giraldo, S., Tobon-Val[illegible]ncia, E., & Eburdy, R. (2020). Identification of critical success factors, risks and opportunities of Indus[illegible]y 4.0 in SMEs. *International Journal of Production Research, 58*(5), 1384–1400. https://d[illegible]g/10.1080/00207543.2019.1636323
Müller, J. M., Buliga, O., & Voigt, K.-I. (2018). Fort[illegible] favo[illegible] the prepared: How SMEs approach business model innovations in Industry 4.0. *Technolog[illegible]cal [illegible]cas[illegible]ng and Social Change, 132*, 2–17. https://doi.org/10.1016/j.techfore.2017.12.019
Nahavandi, S. (2019). Industry 5.0—A human-[illegible]ric s[illegible]lution. *Sustainability, 11*(16), 4371.
Nara, E. O. B., da Costa, M. B., Baierle, I[illegible]., Scha[illegible] J. L., Benitez, G. B., do Santos, L. M. A. L., & Benitez, L. B. (2021). Expected impact of [illegible]ry 4.0 technologies on sustainable development: A study in the context of Brazil's plastic ind[illegible]. *Sus[illegible]able Production and Consumption, 25*, 102–122.
Nimawat, D., & Gidwani, B. D. (2[illegible]21). Identification of cause and effect relationships among barriers of Industry 4.0 using decision-maki[illegible]trial a[illegible]d evaluation laboratory method. *Benchmarking: an International Journal*. https://doi.org/10.11[illegible]J-08-2020-0429/
Raj, A., Dwivedi, G., Sha[illegible] de Sousa Jabbour, A. B. L., & Rajak, S. (2020). Barriers to the adoption of industry 4.0 techn[illegible]logie[illegible] in the manufacturing sector: An inter-country comparative perspective. *International [illegible]rnal o[illegible] Production Economics, 224*, 107546.
Rajput, S., & S[illegible] (2019). Connecting circular economy and industry 4.0. *International Journal of Inform[illegible]ation M[illegible]agement, 49*, 98–113. https://doi.org/10.1016/j.ijinfomgt.2019.03.002
Reinhard[illegible], Oliv[illegible]ra, J. C., & Ring, D. T. (2020). Current perspectives on the development of industry 4.0 i[illegible] the ph[illegible]aceutical sector. *Journal of Industrial Information Integration, 18*, 100131. https://doi.org/10.1016/j.j[illegible].2020.100131
Saa[illegible]. L. (2008). Decision making with the analytic hierarchy process. *International Journal of Services [illegible]ces, 1*(1), 83–98.
[illegible]egal, M. (2018). How automation is changing work. *Nature, 563*(7733), 132–135. https://doi.org/10.1038/d41586-018-07501-y
[illegible]arma, M., & Sehrawat, R. (2020a). A hybrid multi-criteria decision-making method for cloud adoption: Evidence from the healthcare sector. *Technology in Society, 61*, 101258. https://doi.org/10.1016/j.techsoc.2020.101258
Sharma, M., & Sehrawat, R. (2020b). Quantifying SWOT analysis for cloud adoption using FAHP-DEMATEL approach: Evidence from the manufacturing sector. *Journal of Enterprise Information Management, 33*(5), 1111–1152. https://doi.org/10.1108/JEIM-09-2019-0276
Sharma, M., Gupta, R., & Acharya, P. (2020a). Analysing the adoption of cloud computing service: A systematic literature review. *Global Knowledge, Memory and Communication, 70*(1/2), 114–153. https://doi.org/10.1108/GKMC-10-2019-0126

RETRACTED ARTICLE

Sharma, M., Gupta, R., & Acharya, P. (2020b). Factors influencing cloud computing adoption for higher educational institutes in India: A fuzzy AHP approach. *International Journal of Information Technology and Management, 19*(2–3), 126–150.

Sharma, M., Gupta, R., & Acharya, P. (2020c). Prioritizing the critical factors of cloud computing adoption using multi-criteria decision-making techniques. *Global Business Review, 21*(1), 142–161.

Sharma, M., Kamble, S., Mani, V., Sehrawat, R., Belhadi, A., & Sharma, V. (2021). Industry 4.0 adoption for sustainability in multi-tier manufacturing supply chain in emerging economies. *Journal of Cleaner Production, 281*, 125013. https://doi.org/10.1016/j.jclepro.2020.125013

Sharma, M., Sehrawat, R., Luthra, S., Daim, T., & Bakry, D. (2022a). Moving towards industry 5.0 in the pharmaceutical manufacturing sector: Challenges and solutions for Germany. *IEEE Transactions on Engineering Management*. https://doi.org/10.1109/TEM.2022.3143466

Sharma, M., Antony, R., & Tsagarakis, K. (2023a). Green, resilient, agile, and sustainable fresh food supply chain enablers: Evidence from India. *Annals of Operations Research*. https://doi.org/10.1007/s10479-023-05176-x

Sharma, M., Al Khalil, A., & Daim, T. (2022b). Blockchain technology adoption: Multinatio[illegible]nal analy[illegible] of the agriculture supply chain. *IEEE Transactions on Engineering Management*. https://[illegible]oi.org/10.1109/TEM.2022.3193688

Sharma, M., Raut, R. D., Sehrawat, R., & Ishizaka, A. (2023b). Digitalisation of manufa[illegible] operations: The influential role of organisational, social, environmental, and technological im[illegible]diments. [illegible]*pert Systems with Applications, 211*, 118501. https://doi.org/10.1016/j.eswa.2022.11850[illegible]

Skobelev, P. O., & Yu Borovik, S. (2017). On the way from Industry 4.0 to I[illegible]stry 5.[illegible]rom digital manufacturing to digital society. *Industry 4.0, 2*(6), 307–311.

Stegemann, S. (2016). The future of pharmaceutical manufacturing in th[illegible] of the scientific, social, technological and economic evolution. *European Journal of Pharmaceu[illegible] Sciences, 90*, 8–13.

Stentoft, J., Wickstrøm, K. A., Philipsen, K., & Haug, A. (2021). Driv[illegible] barriers for Industry 4.0 readiness and practice: Empirical evidence from small and medium-sized [illegible]facturers. *Production Planning & Control, 32*(10), 811–828.

Stević, Ž, Pamučar, D., Vasiljević, M., Stojić, G., & Kor[illegible] (2017). Novel integrated multi-criteria model for supplier selection: Case study construction [illegible]pany. [illegible]*ymmetry, 9*(11), 279.

Strauss, A., & Corbin, J. (1990). *Basics of qualita[illegible]ve [illegible]arch*. Sage Publications.

Sung, T. K. (2018). Industry 4.0: A Korea per[illegible]ective. *Te[illegible]nological Forecasting and Social Change, 132*, 40–45. https://doi.org/10.1016/j.techfo[illegible].11.0[illegible]5

Teodosiu, C., & Castells, F. (2017). Envi[illegible]nmenta[illegible]gineering and management, progresses and challenges for sustainability: An introductio[illegible] to [illegible]EM08. *Process Safety and Environmental Protection, 108*, 1–6.

Theorin, A., Bengtsson, K., Provos[illegible], Liede[illegible], Johnsson, C., Lundholm, T., & Lennartson, B. (2017). An event-driven manufacturing information system architecture for Industry 4.0. *International Journal of Production Research, 55*(5), [illegible]297–1[illegible]11.

Tseng, M.-L., Islam, M. S., Karia, N., [illegible]zi, F. A., & Afrin, S. (2019). A literature review on green supply chain management: Trends [illegible]ture challenges. *Resources, Conservation and Recycling, 141*, 145–162.

Wan, J., Tang, S., Li, Di., Im[illegible]n, [illegible], Zhang, C., Liu, C., & Pang, Z. (2018). Reconfigurable smart factory for drug packin[illegible] [illegible]ealthc[illegible]re industry 4.0. *IEEE Transactions on Industrial Informatics, 15*(1), 507–516.

Xu, D. L., Xu, [illegible]. (2018). Industry 4.0: State of the art and future trends. *International Journal of Produ[illegible]tion R[illegible]arch, 56*(8), 2941–2962.

Xu, X., Y[illegible], Lu., [illegible]gel-Heuser, B., & Wang, L. (2021). Industry 4.0 and Industry 5.0—Inception, concept[illegible]on and [illegible]ception. *Journal of Manufacturing Systems, 61*, 530–535.

Ya[illegible]av, G., Luthr[illegible], S., Jakhar, S. K., Mangla, S. K., & Rai, D. P. (2020). A framework to overcome sustainable [illegible]pply [illegible]hain challenges through solution measures of industry 4.0 and circular economy: An automotive [illegible] *Journal of Cleaner Production, 254*, 120112. https://doi.org/10.1016/j.jclepro.2020.120112

[illegible]hang, [illegible]M., Guo, H., Huo, B., Zhao, X., & Huang, J. (2019). Linking supply chain quality integration with mass customization and product modularity. *International Journal of Production Economics, 207*, 227–235.

[illegible]eng, T., Ardolino, M., Bacchetti, A., & Perona, M. (2021). The applications of Industry 4.0 technologies in manufacturing context: A systematic literature review. *International Journal of Production Research, 59*(6), 1922–1954.

Zhong, R. Y., Xun, Xu., Klotz, E., & Newman, S. T. (2017). Intelligent manufacturing in the context of industry 4.0: A review. *Engineering, 3*(5), 616–630.

Publisher's Note Springer Nature remains neutral with regard to jurisdictional claims in published maps and institutional affiliations.

RETRACTED ARTICLE
